# Coverage of defects over toes with distally based local flaps: A report of four cases

**DOI:** 10.4103/0970-0358.41114

**Published:** 2008

**Authors:** Ashok Raj Koul, Rahul K. Patil, Vinoth Kumar Philip

**Affiliations:** Department of Plastic and Reconstructive Surgery, Medical Trust Hospital, Cochin - 682 016, Kerala, India

**Keywords:** Distal foot defects, distally based flaps, toe defects

## Abstract

Reconstruction of the distal foot, especially of the toe has always been a challenging problem. Various methods have been tried with variable success rates and limitations. Presented here is a series of four cases, where distally based flaps were used. Two of them were Extensor Digitorum Brevis (EDB) muscle flaps and the other two were first dorsal metatarsal artery (FDMA) based skin flaps. One in each of the two was augmented with a plantar V-Y advancement flap. All flaps survived completely without any flap- or donor site-related complications. The patients were ambulated two weeks following the reconstruction and were symptom-free after an average follow-up of thirteen months. Distal flaps based on the dorsalis pedis system provide a reliable cover for distal foot defects.

## INTRODUCTION

Distal foot reconstruction, especially of wounds over the toes, has always been a challenging problem in plastic surgery. The various reconstruction methods available are skin graft, random pattern flap; cross-leg flap and free flaps but all these methods have their own advantages and limitations. Skin grafts cannot be used on bare bone or tendons. Other flaps may be bulky, unreliable and may need uncomfortable patient positioning or microsurgical techniques. Reverse Dorsalis pedis flaps are usually based on the deep plantar branch of the dorsalis pedis artery (DPA)[1] or on the distal communication between the first dorsal metatarsal system and the plantar system.[2] When flaps are based on connection with the DPA, as against the FDMA, the reach of the vascular pedicle is limited and may not reach the distal foot especially in wounds of the toes. The reverse flaps consisting of either skin or muscles based on the distal communication have a longer vascular pedicle and can be used for coverage of toe defects.[2]

## MATERIALS AND METHODS

Presented here is a series of four cases where distally based flaps on the Dorsalis pedis system have been used for the coverage of toe defects. We have clubbed the distally based muscle flap and the Reverse First dorsal metatarsal artery (FDMA)-based skin flap because their vascular anatomy and applications are similar.

During the period from December 2005 to August 2006, four patients with defects of the toes were managed with distally based local flaps. Patients’ charts were evaluated retrospectively. Age, gender, type of injury, site and dimensions of defects, type of flap used, timing of flap coverage, operative time, duration of hospital stay, complications, flap survival and time of return to normal ambulation were noted.

Soft tissue reconstructions were carried out by a team of three surgeons consisting of a senior plastic surgeon (first author) and two residents. Definitive reconstruction was carried out along with initial debridement whenever possible. The indications for flap reconstruction were the exposure of vital tissues such as tendons, bone joints and size of the defect suitable for local flap cover.

All cases underwent preoperative assessment of vascular viability using physical and Doppler examinations. The relevant vascular anatomy and the surgical technique have been discussed below.

## Relevant arterial anatomy

**Figure 1:**
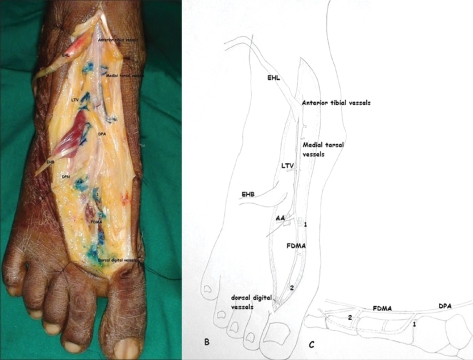
(A) fresh cadaveric dissection of the anatomy of dorsalis pedis artery and its relations (after dissecting out Extensor hallucis longus (EHL), Extensor hallucis brevis (EHB) and the peroneal nerve). (B) Simplified line diagram showing the branches of dorsalis pedis artery C) Schematic sagital section showing the communications between dorsal and plantar systems. ATV: anterior tibial vessels; DPA: dorsalis pedis vessels; FDMA: first dorsal metatarsal artery; AA: arquet artery; LTV: lateral tarsal vessels; 1 communication of DPA/FDMA with plantar arch (lateral plantar artery; 2: communication of FDMA with plantar counterpart

The Dorsalis pedis artery (DPA) is a continuation of the anterior tibial artery passing below the extensor retinaculum (Figure 1A, B and C), it then ends as the first dorsal metatarsal artery (FDMA) after giving off the plantar branch. The Dorsalis pedis artery gives off abundant cutaneous arteries, muscular branches and lateral and medial tarsal branches between the extensor retinaculum and the deep plantar branch.[3] The lateral tarsal artery supplying the extensor digitorum brevis (EDB) serves as the pedicle on which the distally based EDB is elevated. The FDMA courses through the first intermetatarsal space distally in the subcutaneous tissue or deep to or within the first dorsal interosseous muscle.[4] It then passes dorsal to the transverse metatarsal ligament and divides into two dorsal digital arteries to the toes. It communicates with the plantar arterial network *via* the distal communicating artery. The FDMA gives off numerous cutaneous branches to provide enough arterial supply to the intermetatarsal skin.[5] The cutaneous branches from the DPA and the first dorsal metatarsal artery supply the base of the FDMA flap. An adequate arterial inflow can be provided by the distal communicating artery from the plantar system for raising reliable flaps. The blood outflow of the flap relies on the communication between the superficial and deep venous networks.[6]

## Surgical technique

A preoperative Doppler examination evaluates the blood flow of the DPA, the plantar artery and the FDMA and their direction. The FDMA flap is designed so that the DPA and the FDMA are in the middle of the flap. It can be extended from the lower margin of the extensor retinaculum to the first intermetatarsal area and can be safely raised from the whole width of the foot.

A pneumatic tourniquet is applied and the skin incision is started from the proximal side of the flap so that the DPA is exposed. Dissection is continued distally down to expose the deep plantar branch and the FDMA. Care should be taken not to damage the communicating branches.

The EDB is a type II muscle, and a branch from the lateral tarsal artery is the dominant pedicle. The EDB flap is similarly elevated with additional precautions taken not to damage the lateral tarsal artery. The skin on the lateral side of the incision is retracted as a flap along with the long extensor tendons to avoid undermining and to expose the EDB. After identifying and preserving the pedicle, the EDB tendons are divided and as much muscle is taken as needed.

The DPA and the veins in the proximal side of the flap and the deep plantar branch are then blocked with a microvascular clamp and the tourniquet is released. The flap is observed for adequacy of cirulation The flap is allowed to be perfused for about twenty minutes and then the proximal DPA is divided between ligatures. The flap is transposed to cover the defect. The deep plantar branch is ligated and divided only if required to extend the reach of the flap, if the flap is not reaching the defect.

The reverse flap, which is based on the communication between the dorsal and the plantar systems, can be easily transferred to the distal foot. A thick split-thickness graft from the groin or thigh is used to resurface the donor site of the FDMA skin flap. The donor site of muscle can be closed primarily.

## RESULTS

Flaps based on the reverse Dorsalis pedis system were used to reconstruct four defects over the great toes in four male patients [Table 1]. The average age of these patients was 32.5 years (range 27-41 years). All defects were posttraumatic. Two flaps were EDB muscle flaps while the other two were reverse FDMA skin flaps. A plantar V-Y advancement flap was additionally used one in each of these groups to avoid tight closure and to limit the needed flap size and hence, the donor site morbidity.

**Table 1:** Patient-details

*Case*	*Age/sex*	*Defect site*	*Defect size (cm)*	*Flap used*	*Donor site*	*Complication*
I	27/M	Great toe PPX	6*5	Distally based EDB	Closed primarily	Nil
II	40/M	Great toe PPX	3*5.5	Reverse FDMA + Plantar V-Y	Skin grafted	Nil
III	29/M	Great toe ppx	4*3	Reverse FDMA	Skin grafted	Nil
IV	33/M	Dorsum great toe	6*8	Reverse EDB + Plantar V-Y	Closed primarily	Nil

All flaps survived completely without any flap- or donor site-related complications. The average operative time was two hours. Patients were ambulated two weeks following the operation and the mean follow-up period was 13 months. In all patients, the donor and/or recipient site grafts were stable and the patients were comfortable even with shoes without any complications.

## Representative cases

**Figure 2:**
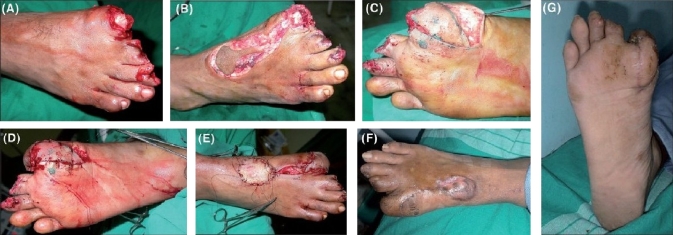
(A) 40/M with defect over great toe. (B) Reverse FDMA flap being elevated. (C) Plantar V-Y advancement. (D) After closure Plantar aspect. (E) Dorsum of foot after graft application. (F) Dorsum: well-settled flap. (G) Plantar aspect: well-settled flap

**Case 1:** In a 40 year-old male with a posttraumatic defect over the great toe [Figure 2A], a distally based FDMA skin flap was elevated [Figure 2B] and a plantar V-Y advancement flap was used for the same defect to assist tension-free closure [Figure 2C]. Figures 2D and E show the dorsal and plantar sides after final closure while Figures 2F and G show the late postoperative, well-settled flaps.

**Figure 3:**
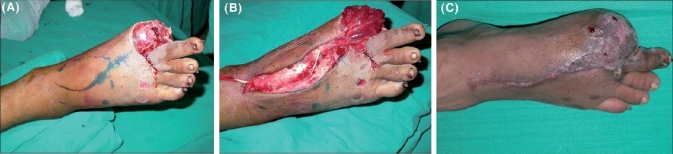
(A) 27/M with defect over great toe. (B) Defect being covered with distally based EDB. (C) Final late postoperative.

**Case 2:** In a 27 year-old male with a defect over the great toe [Figure 3A], a distally based EDB flap was used to cover the defect [Figure 3B]. The late postoperative well-settled flap is shown in Figure 3C.

## DISCUSSION

Soft tissue cover of defects over the toes is a difficult problem because of the thin normal soft tissue which quickly exposes the tendons and bones in case of trauma or infection. As per the reconstructive ladder, primary closure comes as the first option which is difficult in these areas even with minimal soft tissue loss. Skin grafting is again a poor option because of the inadequate recipient bed.[7] Also, the durability of the skin graft over such a bed is questionable as it is subjected to the daily trauma of ambulation. Local random flaps have limited reach and are often unreliable due to local trauma or chronic scarring.[8] The problem gets compounded especially when associated with fractures, osteomyelitis and chronic infection.[9] Pedicled muscle flaps were first advocated by Ger as the next option on the reconstructive ladder.[10] Ger suggested that rotation of such a flap would improve the local blood flow and create a suitable surface for a skin graft. Free flaps are effective for the repair of a wide range of skin defects, however, they warrant the use of special instruments and microsurgical techniques which are not always available.[11]

The dorsalis pedis flap was first described by McGraw and Furlow in 1975.[3] In the following years, it was utilized as a pedicled, island or free flap.[6,12] Only in 1987 was it utilized as a distally based pedicled flap[13] to cover defects of the distal foot, especially after amputation of the toes and/or the other toes.[14,15]

A traditional distally based dorsalis pedis island has been successfully applied in the reconstruction of the defect of a foot after the toes were amputated.[1] When the wound involves the toes, the vascular pedicle based on the deep plantar branch is limited and the distal communication between the FDMA and the plantar system becomes invaluable in these cases.[4,14] Although the traditional reverse dorsal pedis flap can be designed to include the area over the extensor retinaculum and even extend proximally over the extensor retinaculum for 3 cm,[1,16] the FDMA flap is safely limited to below the level of the extensor retinaculum.[17]

The venous outflow is through the communication between the deep and superficial venous systems. It should be emphasized that the FDMA is absent in nearly 15% of all cases,[3,14] so preoperative Doppler flowmetry is mandatory to evaluate the DPA, FDMA and the communications.[18,19] To be sure, we confirm the same by clamping the proximal pedicle for 20 minutes intraoperatively. If the blood inflow and outflow of the flap is sufficient, only then is the proximal pedicle divided and ligated. Although a potential risk in applying these flaps is the insufficient venous drainage, we encountered no problems with blood inflow or outflow.

In 1979, McCraw[13] proposed the use of the EDB muscle as a local option for tissue defects of the foot. Its first clinical application was reported much later in 1985.[20,21] The vascular anatomy of this type II muscle flap[22] being relatively constant makes its use as a proximally or distally based flap reliable.

The size of the EDB muscle can be up to 30 cm^2^ and can cover defects of significant sizes over the toes. It can be used sometimes even in larger defects, as often, only the critical areas and not the full defect need flap cover. This will avoid the lateral dissection near the muscle origin to take the complete muscle and avoid donor site complications resulting from prolonged strong traction of the skin edges.

The main [potential] disadvantage of the reverse FDMA flap is the donor site morbidity. The complications are similar to the reverse dorsalis pedis artery flap like poor take of the skin graft on donor site, pain[18] and ulceration.[3,12,16,18] To reduce the morbidity of the donor site, it is essential and necessary to preserve the peritenon of the exposed tendons on the dorsum of the foot once the flap is elevated. Secondly, the flap size can be reduced to avoid donor site complications. In two of our patients, plantar V-Y advancement was carried out to reduce the defect size.
